# Cord Blood-Derived Macrophage-Lineage Cells Rapidly Stimulate Osteoblastic Maturation in Mesenchymal Stem Cells in a Glycoprotein-130 Dependent Manner

**DOI:** 10.1371/journal.pone.0073266

**Published:** 2013-09-12

**Authors:** Tania J. Fernandes, Jason M. Hodge, Preetinder P. Singh, Damien G. Eeles, Fiona M. Collier, Ian Holten, Peter R. Ebeling, Geoffrey C. Nicholson, Julian M. W. Quinn

**Affiliations:** 1 Northwest Academic Centre, Department of Medicine, The University of Melbourne, Victoria, Australia; 2 Barwon Biomedical Research, The Geelong Hospital, Geelong, Victoria, Australia; 3 School of Medicine, Deakin University: Barwon Health, Geelong, Victoria, Australia; 4 Prince Henry's Institute of Medical Research, Clayton, Victoria, Australia; 5 Department of Human Biosciences, La Trobe University, Bundoora, Victoria, Australia; 6 Department of Plastic Surgery, Barwon Health, Geelong, Victoria, Australia; 7 Rural Clinical School, The University of Queensland, Toowoomba, Queensland, Australia; 8 Department of Biochemistry and Molecular Biology, Monash University, Clayton, Victoria, Australia; INSERM U1059/LBTO, Université Jean Monnet, France

## Abstract

In bone, depletion of osteoclasts reduces bone formation *in vivo*, as does osteal macrophage depletion. How osteoclasts and macrophages promote the action of bone forming osteoblasts is, however, unclear. Since recruitment and differentiation of multi-potential stromal cells/mesenchymal stem cells (MSC) generates new active osteoblasts, we investigated whether human osteoclasts and macrophages (generated from cord blood-derived hematopoietic progenitors) induce osteoblastic maturation in adipose tissue-derived MSC. When treated with an osteogenic stimulus (ascorbate, dexamethasone and β-glycerophosphate) these MSC form matrix-mineralising, alkaline phosphatase-expressing osteoblastic cells. Cord blood-derived progenitors were treated with macrophage colony stimulating factor (M-CSF) to form immature proliferating macrophages, or with M-CSF plus receptor activator of NFκB ligand (RANKL) to form osteoclasts; culture medium was conditioned for 3 days by these cells to study their production of osteoblastic factors. Both osteoclast- and macrophage-conditioned medium (CM) greatly enhanced MSC osteoblastic differentiation in both the presence and absence of osteogenic medium, evident by increased alkaline phosphatase levels within 4 days and increased mineralisation within 14 days. These CM effects were completely ablated by antibodies blocking gp130 or oncostatin M (OSM), and OSM was detectable in both CM. Recombinant OSM very potently stimulated osteoblastic maturation of these MSC and enhanced bone morphogenetic protein-2 (BMP-2) actions on MSC. To determine the influence of macrophage activation on this OSM-dependent activity, CM was collected from macrophage populations treated with M-CSF plus IL-4 (to induce alternative activation) or with GM-CSF, IFNγ and LPS to cause classical activation. CM from IL-4 treated macrophages stimulated osteoblastic maturation in MSC, while CM from classically-activated macrophages did not. Thus, macrophage-lineage cells, including osteoclasts but not classically activated macrophages, can strongly drive MSC-osteoblastic commitment in OSM-dependent manner. This supports the notion that eliciting gp130-dependent signals in human MSC would be a useful approach to increase bone formation.

## Introduction

Osteoblasts are specialised bone forming cells that derive from local mesenchymal progenitors through multi-step commitment and maturation processes that are dependent on transcription factors Runx2 and osterix [1]. Such progenitors include MSC populations found in bone and in extraosseous tissues [2], highly proliferative cells expressing CD73, CD90 and CD105 but lacking hematopoietic markers [3], [4]. When purified, these cells can form functional osteoblasts both *in vitro* and *in vivo* [5]–[7] but also have the capacity to form other types of stromal cells, such as adipocytes [2], [7], [8]. While the MSC transition to osteoblasts is not fully understood, the appropriate enhancement of such a process might form the basis of therapies that increase bone formation in patients with low bone mass.

Maintenance of bone strength requires bone remodelling, whereby old or damaged bone is removed by osteoclasts (multinucleated bone resorbing cells of the myelomonocytic lineage) [9]–[11] and the bone removed by osteoclasts subsequently replaced by osteoblast action. This, and the impairment of bone formation following anti-osteoclastic therapies [12], suggest a functional link between osteoclast and osteoblast activity. However, osteoclast stimulation of mature osteoblast activity has not been convincingly demonstrated. An alternative possibility is that osteoclasts exert their influence on osteoblast progenitors, such as local MSC. Indeed, MSC are chemotactically attracted to bone sites undergoing remodelling [13]. It is notable, however, that, not all bone formation requires osteoclast action; osteal macrophages, a locally resident CD11b^+^F4/80^+^ cell population closely related to osteoclasts, also exert a significant regulatory influence on bone formation [14], [15]. Osteal macrophages are located in close proximity to osteoblasts and their removal greatly decreases bone formation [16], but the nature of their influence on osteoblasts is also unclear. Macrophages display many diverse functions central to innate immunity and adaptive immune responses, especially via antigen presentation and cytokine production, but also play regulatory and cytokine secretory roles in many tissues. Macrophages respond to environmental stimuli by altering their behaviour and excitation states, notably their phenotype can be polarised by Th_1_-cytokines towards classical activation and by Th_2_-cytokines towards a number of alternative activation states. An abundance of classically activated macrophages in the bone, typically seen in inflammatory joint diseases, is generally associated with impaired bone formation, perhaps related to suppression of osteoblastic Wnt responses [17]. Therefore, understanding the influence of macrophages in different activation states on immature osteoblast-lineage cells is of great interest in bone biology.

We have previously employed cord blood-derived immature myelomonocytic-lineage cells rich in colony forming units (CFU)-GM as an excellent source of human osteoclast-forming cells [18], [19]. Such cord blood mononuclear cells contain populations broadly similar to immature myelomonocytic populations found in bone marrow. They are a rich source of immature macrophages that proliferate with M-CSF or GM-CSF treatment [18], [20] and, when treated with M-CSF plus RANKL, they form large numbers of bone resorbing osteoclasts. Circulating adult CD14^+^ monocytes also form osteoclasts with RANKL/M-CSF stimulus but do so far more slowly and with much lower yield [20], [21], reflecting their preponderance of mature cells. We therefore employed the superior cord blood-derived progenitors to generate both macrophages and osteoclast-rich cultures to study their effects on osteoblastic differentiation in human MSC. A related approach was employed in the recent work of Guihard *et al.* [22] and Nicolaidou *et al.* [23] who both found that CD14^+^ adult monocytes enhanced osteoblastic differentiation of MSC in a manner at least partly dependent on the IL-6 family cytokine oncostatin M (OSM). Their observations differed in certain key respects, however. Guihard *et al.* [22] found that medium conditioned by CD14^+^ (especially classically activated CD14^+^ cells) strongly enhanced MSC maturation, while Nicolaidou *et al.* [23] observed macrophage-MSC contact was essential for such pro-osteoblastic activity. While these are seminal pieces of work, clearly further studies are needed that employ other macrophage lineage cells that MSC encounter in bone. In our studies we found that both cord-blood derived macrophage and osteoclast populations produce soluble factors that very rapidly (within 4 days) drive osteoblastic maturation in these MSC populations. This activity was dependent upon OSM secretion but neither cell contact nor classical activation (in macrophages) was required for osteoblastic maturation of MSC. This provides further evidence for the role of osteoclasts, macrophages and OSM in the regulation of bone metabolism.

## Materials and Methods

### Ethics Statement

Human umbilical cord blood and adipose tissue samples were obtained with informed, written consent from healthy donors under protocols approved by Barwon Health Human Research and Ethics Committee.

### Cell media and reagents

Eagle's minimum essential medium (MEM), Dulbecco's Modified Eagle's Medium (DMEM), penicillin/streptomycin solutions, paraformaldehyde, Fast Garnet GBC, naphthol AS-BI-phosphate, collagenase-type-1, p-nitrophenylphosphate, p-nitrophenyl, diethanolamine, Alizarin Red, cetylpyridinium chloride, dexamethasone, dimethyl sulphoxide (DMSO) and 3-(4,5-Dimethylthiazol-2-yl)-2,5-diphenyltetrazolium bromide, insulin, 3-Isobutyl-1-methylxanthine (IBMX) and indomethacin were purchased from Sigma-Aldrich (St. Louis, USA). β-glycerophosphate disodium salt was purchased from Merck Millipore (Kilsyth, Australia). L-Ascorbic acid phosphate was purchased from NovaChem Pty Ltd (Melbourne, Australia). Non-essential amino acids (100X) and fetal bovine serum (FBS) were purchased from Bovogen (Melbourne, Australia). Human oncostatin-M (OSM) ELISA, anti-OSM and anti-gp130 blocking monoclonal antibodies and recombinant OSM, Wnt3A, GM-CSF, IL-4, interferon-γ (IFNγ) and BMP-2 proteins were purchased from R&D Systems (Minneapolis, USA). Ready-SET-Go^®^ human TNF, IL-1β and IL-10 ELISAs were obtained from eBioscience (San Diego, CA). Ficoll-Paque was purchased from GE Healthcare Life Sciences (Rydalmere, Australia). MethoCult GF H4534 (Iscove's medium containing 1% methylcellulose, 30% FBS, 1% bovine serum albumin, 10^−4^M 2-mercaptoethanol, 2 mM L-glutamine, 10 ng/mL recombinant human GM-CSF, 10 ng/mL IL-3, and 50 ng/mL stem cell factor) was purchased from Stem-Cell Technologies (Tullamarine, Australia). Soluble RANKL^158–316^-GST fusion protein (RANKL) was produced in-house from a construct kindly supplied by Dr. F. Patrick Ross (Hospital for Special Surgery, NY) as previously described [24]. All other reagents were analytical grade.

### Generation of macrophages and osteoclasts

Collection of human umbilical cord blood, isolation of a mononuclear cell fraction, expansion of CFU-GM-derived osteoclast precursors and differentiation of mature human osteoclast have been previously described [18]. Briefly, cord blood mononuclear cell fraction (CBMC) was isolated by Ficoll-Paque density gradient centrifugation and the cells (3×10^6^ cells/culture) were suspended in 3.0 mL Methocult GF H4534 in 35 mm diameter (6-well) plates and incubated at 37°C in humidified atmosphere of 5% CO_2_-air for 11 days to generate CFU-GM colonies (>80%) and CFU-M colonies (5–10%); hereafter these CBMC-derived populations are referred to as CFU-GM, as previously described [19]. These cell populations were pooled in PBS, centrifuged, and resuspended in DMEM containing 10% heat inactivated (55°C for 30 minutes) FBS, non-essential amino acids, penicillin 50 U/mL; streptomycin 50 mg/mL and 2 mM L-glutamine (DMEM/FBS) and then cultured (7×10^6^ cells/175 cm^2^ flask) for 14 days with M-CSF (25 ng/mL) alone to generate proliferating macrophages, or M-CSF and RANKL (125 ng/mL) to generate osteoclasts.

To generate classically activated macrophages [25] CFU-GM (7×10^6^ cells/175 cm^2^ flask) were cultured in MEM/FBS with one of the three following stimulations: (i) GM-CSF (10 ng/mL) for 20 days, (ii) GM-CSF (10 ng/mL) for 14 days, followed by GM-CSF (10 ng/mL) and interferon-gamma (IFNγ) (1?? ng/mL) for 6 days, or (iii) GM-CSF (10 ng/mL) for 14 days, followed by GM-CSF (10 ng/mL) and IFNγ (100 ng/mL) for 3 days, then GM-CSF (10 ng/mL) and lipopolysaccharide (LPS; 100 ng/mL) for 3 days. To generate macrophages undergoing alternative activation, CFU-GM (7×10^6^ cells/175 cm^2^ flask) were cultured in MEM/FBS with M-CSF (25 ng/mL) for 11 days, then M-CSF (25 ng/mL) and IL-4 (100 ng/mL) for 6 days.

### Confirmation of macrophage and osteoclast identity

Adherent macrophages were identified by α-napthyl acetate esterase (non-specific esterase; NSE) histochemistry. Cell were fixed in 4% paraformaldehyde for 10 mins, then incubated in Fast Blue BB-based substrate solution prepared from a commercial kit as per manufacturer's instructions (Sigma-Aldrich, Catalogue number 91A-1KT). Cells were also scraped from the culture surface and incubated with phycoerythrin labelled anti-CD14 (anti-CD14-PE) or anti-CD16-FITC (BD Australia, North Ryde, Australia) antibodies and analysed by flow cytometry as below. Osteoclasts were identified by tartrate-resistant acid phosphatase (TRAP) expression and multinuclearity (>2 nuclei) [26]. To confirm the formation of functional osteoclasts, CFU-GM were seeded (4×10^4^/well) into 6 mm diameter tissue culture wells (96-well tissue culture plates) containing 28.3 mm^2^ slices of sperm whale dentine prepared as previously described [25] and cultured in 200 µL DMEM/FBS plus M-CSF (25 ng/mL) and RANKL (125 ng/mL) for 14 days, with medium and mediators replaced twice weekly. After 14 days the cells were fixed in 1% formalin and histochemically reacted to confirm TRAP expression, then cells were removed from dentine slices by brief sonication in chloroform:methanol 2∶1. Xylene-free black ink was applied to the resorbed surface of each slice and residual ink removed by wiping the dentine surface against absorbent paper, leaving resorption pits stained black for assessment by transmission light microscopy [18].

### Conditioned medium collection

**Figure 1:**
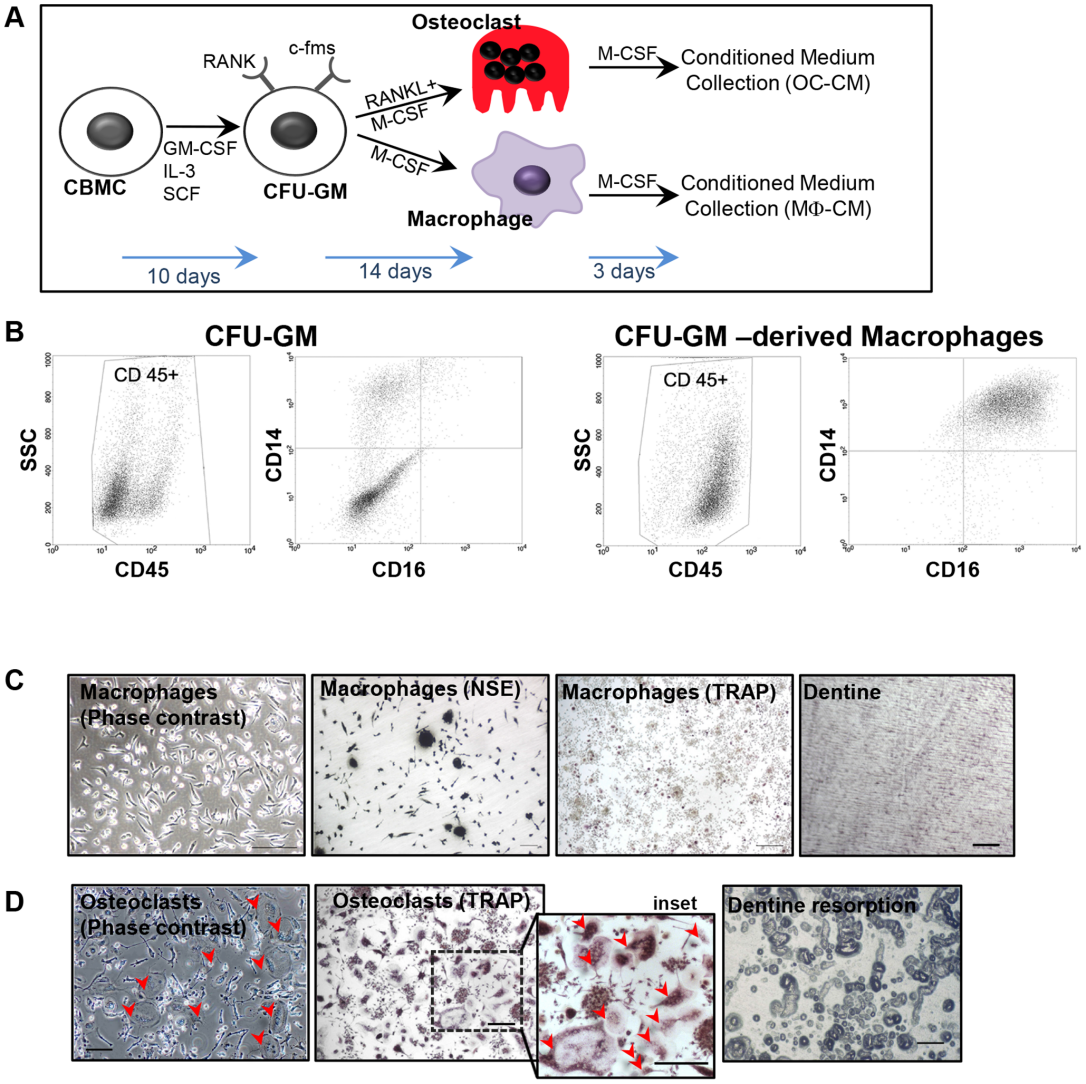
Generating and characterising CFU-GM and CFU-GM-derived populations. (A) Schematic of CM generation as outlined in Materials and Methods: CFU-GM expanded from cord blood mononuclear cells (by incubation for 10 days in semi-solid medium and growth factors) were used to generate osteoclast and macrophage populations. CM from these cells were then used to treat MSC in experiments. (B) CFU-GM and macrophage populations (after CM collection) were analysed for CD45 expression by flow cytometry and these CD45^+^ cells examined for CD14 and CD16 expression. (C) Representative photomicrographs of macrophage cultures showing phase contrast image (plastic substrate), nonspecific esterase (NSE) histochemical staining (dentine substrate), TRAP histochemical staining (dentine substrate), and dentine substrate of macrophage culture after the macrophages have been removed to reveal a complete lack of pit formation. (D) Photomicrographs of osteoclastic cultures, showing phase contrast image (plastic substrate, red arrowhead indicating osteoclasts), TRAP histochemical staining (dentine substrate, including expanded view with osteoclasts indicated by red arrowhead), and dentine substrate of osteoclast cultures after the osteoclasts were removed, which has been stained to reveal extensive pit formation. All scale bars  =  100 µm.

After 14 days culture in either M-CSF (25 ng/mL), or M-CSF (25 ng/mL) and RANKL (125 ng/mL) to produce macrophages and osteoclasts respectively, cells were cultured for a further 3 days in DMEM/FBS with M-CSF (25 ng/mL) (Fig. 1A). This macrophage (MΦ-CM) and osteoclast conditioned medium (OC-CM) was filtered (0.22 µm pore size filters; Corning, Lowell, MA) and stored at −80°C until further use. For activated macrophages, conditioned medium was collected from the last 3 days from each of the stimulated culture conditions. Cell culture medium containing the same mediators was also incubated in the absence of cells for 3 days and this aged medium subsequently used as the experimental control medium.

### Isolation and culture of adipose tissue-derived MSC

Human adipose tissue was collected during elective surgery. To isolate MSC, tissue was teased from blood vessels, minced with a scalpel blade, and digested for 30–45 min with 0.075% collagenase at 37°C with gentle agitation. Enzyme activity was neutralised with basal medium (DMEM containing 10% FBS and penicillin 50 U/mL; streptomycin 50 mg/mL) and the cells were centrifuged at 1200×*g* for 10 mins, resuspended and filtered through a 100 µm cell strainer to remove remaining tissue debris. Cells were pelleted by centrifugation and seeded (10^6^ cells) in tissue culture flasks in basal medium, then incubated at 37°C in humidified atmosphere of 5% CO^2^-air. Cells were passaged by treatment with 0.025% trypsin/EDTA in PBS and diluted 1∶10 in DMEM/FBS. MSC derived from individuals (unpooled) were employed in assays after 5 passages.

### Differentiation of MSC in medium containing osteogenic (OSG) factors

MSC (10^4^ cells/well) were seeded in 6 mm diameter culture wells in DMEM/FBS and cultured overnight. For MSC differentiation time-course, cells were then cultured in osteogenic medium (DMEM/FBS containing dexamethasone (100 nM) β-glycerophosphate (10 mM) and ascorbate-2-phosphate (100 mM)) and assessed for ALP activity and matrix mineralisation at 7, 14 and 21 days of incubation. For conditioned medium experiments, MSC (10^4^ cells/well) were seeded in 6 mm diameter culture wells and cultured in 50% conditioned medium plus 50% osteogenic medium; final concentrations in MSC cultures of dexamethasone β-glycerophosphate and ascorbate-2-phosphate were thus 50 nM, 5 mM and 50 mM respectively. Cells were assessed for ALP activity at 4 days and matrix mineralisation at 14 days or as indicated. For antibody neutralisation assays, MSC were cultured in 50% conditioned medium plus osteogenic medium containing (final concentration) 1 µg/mL anti-gp130 or 10 µg/mL anti-OSM monoclonal antibodies or mouse IgG_1_ control, then assessed for ALP at 4 days and matrix mineralisation and metabolic activity by MTT assay at 14 days.

### MTT metabolic activity assay

After culture, media was completely removed from appropriate wells and MTT solution containing 1.2 mM 3-(4,5-Dimethylthiazol-2-yl)-2,5-diphenyltetrazolium bromide in DMEM was added, and then incubated for 37°C for 4 hours. The supernatant was completely removed and the cells containing the formazan product were solubilised in DMSO for 30 mins. The solubilised solution was transferred to a fresh 96-well plate and optical density (OD) measured at 570 nm using a Tecan Genios Pro photospectrometer.

### Alkaline Phosphatase (ALP) activity assay

To determine cellular ALP activity, cells were lysed in 0.1% Triton X-100 for 30 m at room temperature. A pre-warmed solution containing 10 mg/mL p-nitrophenylphosphate (pNPP) in 10% v/v diethanolamine buffer; 0.5 mM MgCl_2_ pH 9.8 was then added to the lysates and optical density of samples were assessed using a Tecan Genios Pro photospectrometer, OD measured at 410 nm at 37C. This was measured at 2.5 min intervals for 30 mins. Results were converted to standard international units (SIU), equivalent to the conversion by ALP of 1 mM of pNPP to p-nitrophenyl (pNP) per minute. A standard curve was generated by serially diluting 1 mM pNP in diethanolamine buffer and data presented as relative SIU.

### Quantification of *in vitro* matrix mineralisation

MSC were fixed in 1% formalin for 30 minutes and then treated with 40 mM Alizarin Red (ALZ) for 15 min at RT. For quantification of staining, a protocol was adapted from that described by Stanford *et al*. [27]. Cells were washed repeatedly with distilled water and the stain was then solubilised in 3% cetylpyridinium chloride (CPC) in 20 mM sodium phosphate buffer for 45 min. The solubilised supernatant was transferred to a new 6 mm diameter wells and OD measured at 570 nm using a Tecan Genios Pro photospectrometer. A standard curve was generated for ALZ by serially diluting 1 mM ALZ in 3% CPC. OD readings were converted to CaCl_2_ mg/well. CaCl_2_ per well is based on molar equivalent of ALZ to Ca (1∶1); 1 mM (mmol/L) is equivalent to 22.196 ng/L CaCl_2_

### Flow cytometric analysis

Flow cytometry analyses of adipose tissue derived cells, CFU-GM and CFU-GM-derived cells were performed using FacsCalibur and CELLquest software (Becton Dickinson, NJ). Approximately 1×10^5^ cells from each population were labelled with appropriate phycoerythrin (PE), fluoroscein isothyocyanate (FITC) and peridinin-chlorophyll protein (PerCP) labelled antibodies including anti-CD45-FITC, anti-CD14-PE, anti-CD16-FITC, anti-CD34-PE, anti-CD-34–PerCP, anti-CD86-PercP, anti-CD206-FITC, anti-CD73-PE, anti-CD90-FITC (BD Australia, North Ryde, Australia) and anti-CD105-FITC (Abcam plc, Cambridge, UK) according to manufacturer instructions. As negative controls for the fluorescent cell labelling we employed appropriate isotype controls for the antibodies employed. including anti-IgG-FITC, anti-IgG-PE, anti-IgG-PerCP (BD Australia) or anti-IgG2a-FITC (Abcam).

### Real time RT-PCR and semiquantitative RT-PCR analysis of mRNA expression

Cellular RNA was isolated by lysing cells in Trizol and using the illustra RNAspin Mini Kit (GE Healthcare, Melbourne Australia). RNA concentration was determined by spectrophotometer (Nanodrop ND1000). cDNA was synthesized from RNA using the Superscript® III First Strand Synthesis SuperMix system (Life Technologies) as per manufacturer's instructions. To quantify the expression of human Runx2, osterix and OSM mRNA levels we employed real-time PCR analysis of cDNA performed in a 7500 Fast Real-Time PCR System (Applied Biosystems), using TaqMan® Gene Expression Assays using standard commercially available primer/probe mixtures (Applied Biosystems catalogue Hs00231692_m1, Hs01866874_s1 and Hs00171165_m1 respectively). Relative gene expression units were determined using the formula 2^−ΔCt^×1000, where ΔCt values represent the difference between the Ct of the gene of interest and β-actin. For analysis of IL-6, TNF and the classical parathyroid hormone (PTH) receptor (PTH1R) mRNA expression real time PCR analysis (Stratagene Mx3000P) of cDNA was performed using Platinum^®^ SYBR^®^ Green qPCR supermix UDG (Invitrogen) according to manufacturer's instructions and the following conditions: 1 cycle 10 mins 95°C; 40 cycles 30 seconds at 95°C, 1 min at 60°C, 30 seconds at 72°C; 1 cycle 1 min at 95°C, 30 seconds at 55°C, 0 seconds at 95°C) normalized to hypoxanthine phosphoribosyltransferase (HPRT). Primer oligo nucleotide sequences used for real time RT-PCR using the SYBR^®^ Green qPCR-based method were as follows:

HPRT (GenBank accession NM_000194.2) 5′-GACCAGTCAACAGGGGACAT-3′, reverse 5′-CGACCTTGACCATGTTTGGA-3′;

Human TNF (GenBank accession NM_000594.3) forward 5′- ATCTTCTCGAACCCCGAGTGA-3′, reverse 5′- CGGTTCAGCCACTGGAGC T-3′;

Human IL-6 (GenBank NM_000600.3) forward 5′- AAATTCGGTACATCCTCGACGG-3′, reverse 5′- GGAAGGTTCAGGTTGTTTTCTGC-3′;

Human PTH1R (GenBank accession NM_000316.2) forward 5′- ACCTGCACAGCCTCATCTTCA-3′, reverse 5′- CACACAGCCACGAAGACAGC-3′.

To assess human BSP mRNA expression we employed semi-quantitative RT-PCR analysis. cDNA was prepared as above and PCR reactions employed KAPA2G Robust Hotstart PCR kits (KAPA Biosystems, Woburn, MA) according to manufacturer's instructions using an Applied Biosystems Veriti thermal cycler machine (Life Technologies, Carlsbad, CA). Glyceraldehyde-3 phosphate dehydrogenase (GAPDH) mRNA was used as a housekeeping gene expression reference. For BSP the following conditions were used: after 10 mins at 95°C, we used 32 thermal cycles (30 seconds at 95°C, 30 seconds at 58°C, 60 seconds at 72°C) followed finally by 10 min at 72°C. GAPDH analysis used similar conditions with 30 thermal cycles. PCR generated products were separated by electrophoresis on a 1.5% agarose gel containing Sybr Safe^®^ DNA stain (Invitrogen) then visualised under ultraviolet light using a Biorad (Gladesville, Australia) Gel Doc™ 2000 imaging system. Primer oligo nucleotide sequences used for semi-quantitative RT-PCR were as follows:

BSP (GenBank accession NM_004967.3; IBSP) forward 5′- CCTTCTCTGCCCTCTCACTCC-3′, and reverse 5′- ATGAGTCACTACTGCCCTGAAC-3′, product size of 205 base pairs;GAPDH (GenBank accession NM_001256799.1) forward 5′- CACTGACACGTTGGCAGTGG -3′ and reverse 5′- CATGGAGAAGGCTGGGGCTC -3′, product size 405 base pairs.

### Measurement of BMP and canonical Wnt activity

To detect BMP and Wnt activity in conditioned medium we employed luciferase reporter transfection assays. BMP-response element (BMP-RE) [28] and TOPflash TCF/LEF (with a β-catenin-sensitive promoter to detect canonical Wnt signals) Upstate Biotechnology, NY) reporter construct DNA (0.1 µg/well) was co-transfected with pRL Renilla luciferase construct (0.1 µg/well; Promega), into UMR106.01 osteoblast-like cells [29] with Fugene^®^ 6 transfection reagent (Promega) according to manufacturer instructions. Cell cultures were treated in triplicate with CM for 24h, PBS rinsed then lysed wth Passive Lysis Buffer (Promega) for 24h at 4°C. Lysates were transferred to a white flat bottomed 96 well microplate (Corning, Lowell, MA) and signal measured using firefly Luciferase substrate and Stop and Glo^®^ reagents (Promega) as per manufacturer instructions using a EnVision multilabel (PerkinElmer, Waltham, MA) plate reader.

### Statistical analyses

Data are expressed as the mean ± SEM where applicable. Differences between groups were determined using either Student's t-test (for 2 way comparison), or by one-way ANOVA or two-way ANOVA (GLM), followed by Tukey's *post hoc* test as indicated. Statistical significance is indicated thus: * p<0.05, ** p<0.01, *** p<0.001. Statistical significance indicated on graphs is relative to control cultures or between groups connected with capped line.

## Results

### Characterisation of cell populations

*Macrophages*: Flow cytometric analysis showed that most M-CSF expanded CFU-GM cells expressed high levels of leukocyte common antigen (CD45) and 98% expressed CD14 (Fig. 1B, Table 1), confirming them as macrophages. A large proportion of these cells were also CD14^+^/CD16^+^ (Table 1), resembling the ‘non-classical’ subpopulation of monocytes [30], [31]. A majority of these macrophages (81% and 72% respectively) expressed antigen presentation co-stimulatory molecule CD86 and mannose receptor CD206 (Table 1). Populations of macrophages generated by treating cord blood-derived CFU-GM with M-CSF for 14 days expressed NSE but little or no TRAP activity, and did not resorb dentine substrate (Fig. 1C). Conditioned medium from these macrophages showed no detectable human IL-1β and TNF (ELISA, data not shown), while IL-10 levels were detectable but generally low (83.2±12.2pg/ml, mean±SEM).

**Table 1:** Characterisation of CFU-GM and CFU-GM-derived M-CSF-dependent macrophages by flow cytometry.

	CFU-GM Cells	M-CSF Treated Cells
CD45^+^	57.46.42±0.17%	94.94±0.38%
CD14^+^	35.42±0.39%	98.03±0.33%
CD16^+^	6.77±0.61%	46.28±1.06%
CD14^+^ /CD16^+^	6.92±0.51%	45.95±0.39%
CD14^+^ /CD86^+^	0%	81.85±0.92%
CD206^+^	31.92±0.29%	71.89±0.86%
CD14^+^ /CD206^+^	28.84±0.27%	72.89±0.40%
CD34^+^	3.70±1.85%	1.92±1.21%

CFU-GM cells taken immediately after their expansion in semi-solid medium were fluorescently stained by primary labelled antibodies for CD45, CD14, CD16, CD34, CD86 and CD206, and the proportion of positive cells estimated. These cells were compared with macrophages generated from CFU-GM cells by treatment with M-CSF (17 days); two independent experiments analysed in triplicate, mean ±SEM shown.

*Osteoclasts*: Multinucleated osteoclasts were generated by treating CFU-GM with M-CSF and RANKL for 14 days. All cells in these cultures expressed TRAP, and produced extensive resorption pits, demonstrating their functional osteoclastic status (Fig. 1D).

**Figure 2:**
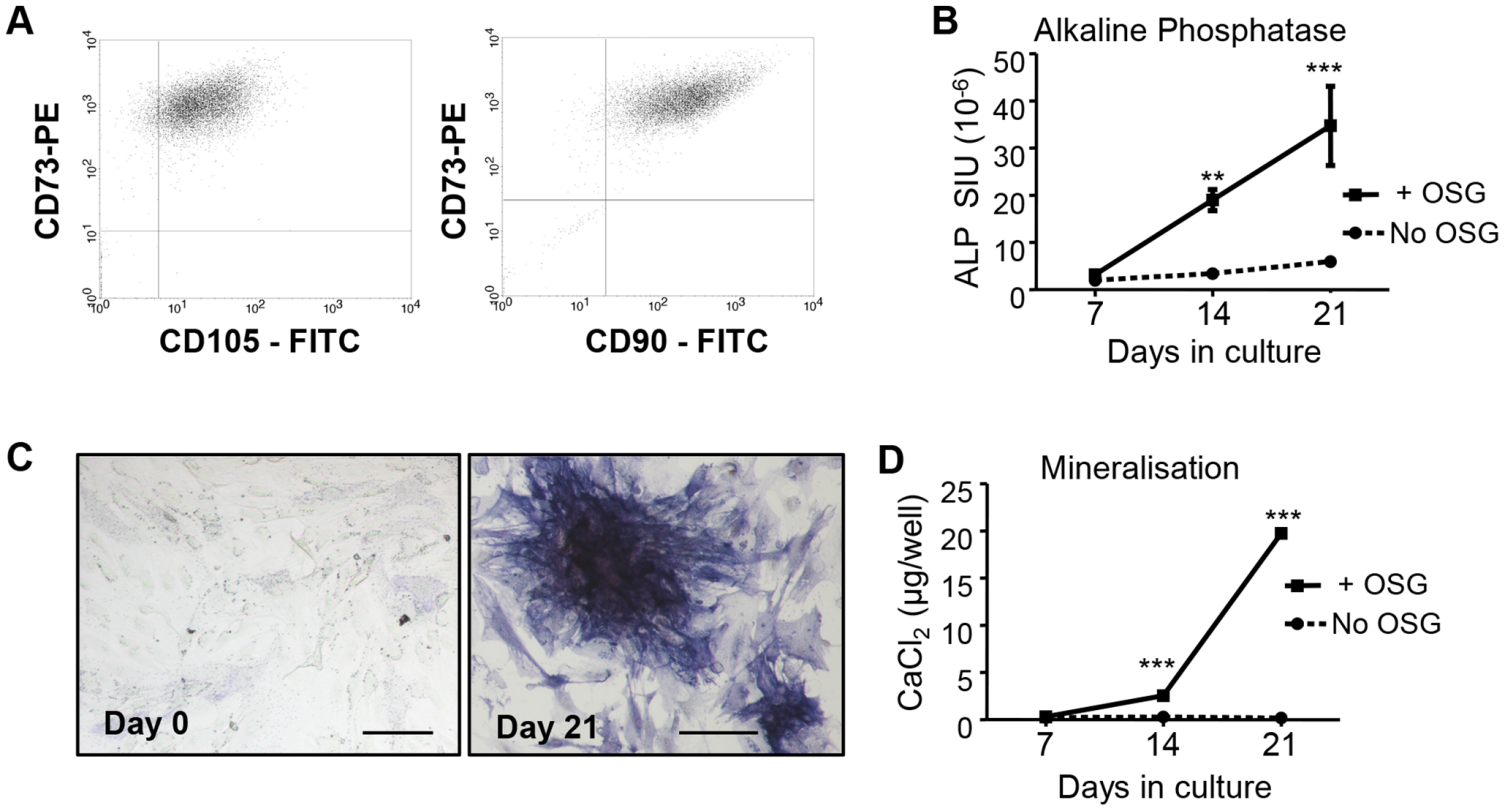
Characterisation of MSCs and their differentiation. (A) FACS analysis of expanded adipose stromal cells in culture revealed a high proportion of cells expressing CD90, CD73 and CD105, indicating a population enriched in MSC; PE  =  phycoerythrin label, FITC  =  fluoroscein isothiocyanate label. To assess their osteogenic capacity, cells were cultured in medium with or without osteogenic factors (OSG; ascorbate, dexamethasone and β-glycerophosphate) for 7, 14 and 21 days and assessed (B) for ALP activity; (C) Photomicrograph ALP histochemical staining of MSC cultures after 0 and 21 days of OSG stimulus, the latter showing clusters of strongly ALP positive (blue) cells; bars  = 100 µm. (D) The ability of the cells to form mineralised matrix was determined by Alizarin Red binding assay. Data displayed as mean ± SEM with statistical significance determined by two-way ANOVA, General Linear Model, and Tukey's *post hoc* test, n = 6, **p≤0.01, ***p≤0.001 relative to respective (no OSG) controls.

*MSC*: MSC populations were isolated from adipose tissue for long term culture. Flow cytometric analysis indicated that MSC populations passaged >5 times contained >95% of cells that were CD73^+^, CD90^+^ and CD105^+^ (Fig. 2A). CD45^+^ cells were not detected, indicating that leukocytes were not present. Cells were seeded 10^4^ cells/6 mm diameter culture well for long term culture. The OB-forming potential of MSC (after 5 passages) was confirmed by culture in osteogenic medium; these cells expressed little ALP by day 14, but expressed high levels by day 21 (Fig. 2B,C). MSC cultures in osteogenic medium formed only low levels of mineralised matrix by day 14 but very high levels by day 21 (Fig. 2D). MSC formed large numbers of adipocytes stained by Oil Red O when incubated with a standard adipogenic stimulus (1 µM dexamethasone plus 175 nM insulin, 450 µM IBMX and 100 µM indomethacin) for 14 days (data not shown), confirming the multipotential nature of these MSC populations.

### Effects of macrophage- and osteoclast-conditioned media on osteoblastic differentiation of MSC

**Figure 3:**
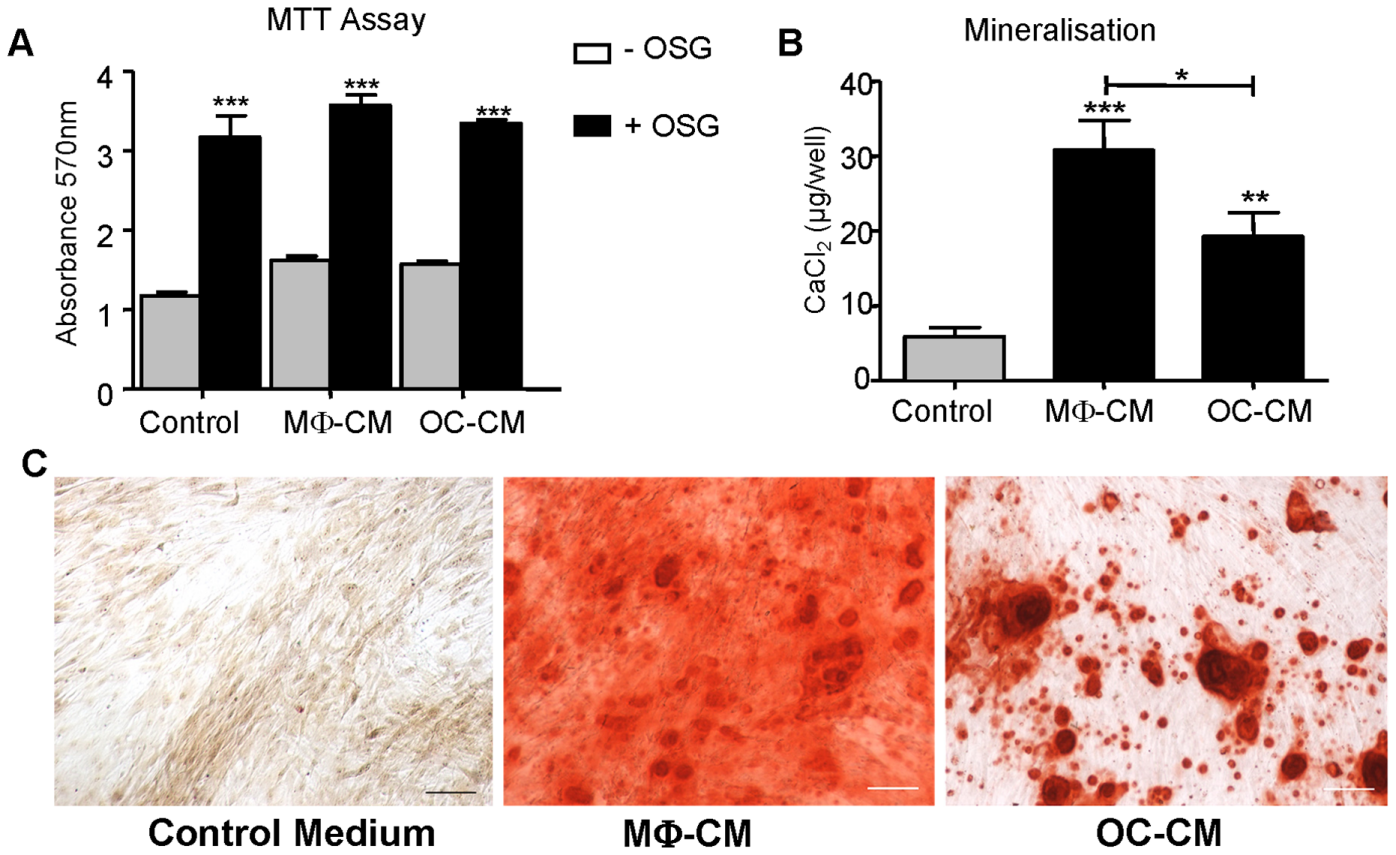
The effects of MΦ-CM and OC-CM on MSC mineralisation and metabolic activity. 10^4^ MSC were cultured in 50% OC-CM or MΦ-CM with osteogenic factors for 14 days. (A) Metabolic activity of cultures assessed by MTT assay. (B) Matrix mineralisation, assessed by quantification of bound Alizarin Red, was greatly enhanced by OC-CM or MΦ-CM treatment of MSC cultured in osteogenic medium. (C) Representative photomicrographs of stained cultures from B; scale bars  = 100 µm. Data displayed as mean ± SEM. Statistical significance relative to controls (adjoining grey columns) determined by Two-Way ANOVA, GLM (A) and One-Way ANOVA, Tukey's *post hoc* test (B), n = 6, *p≤0.05, ***p≤0.001 difference from control medium or as indicated with capped line. Control  = 50% medium conditioned in the absence of cells.

**Figure 4:**
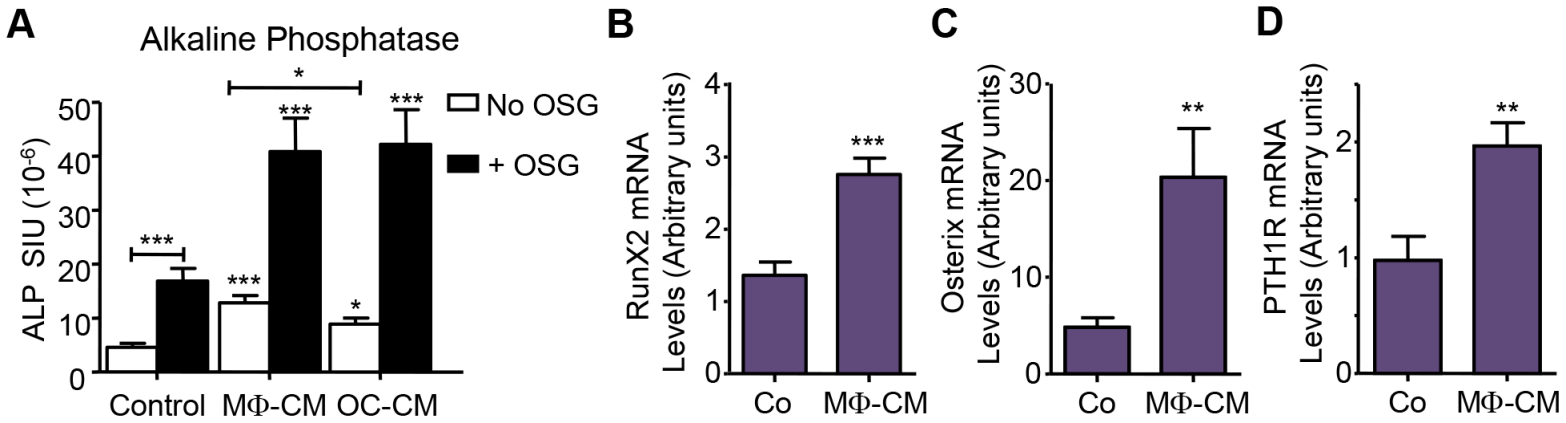
MΦ-CM and OC-CM stimulate MSC osteoblastic maturation. 10^4^ MSC were cultured with 50% OC-CM, MΦ-CM or control medium as noted with osteogenic factors for 14 days and osteoblastic differentiation characteristics (other than matrix mineralisation) were assessed. (A) ALP activity in CM-treated MSC in the presence or absence of osteogenic factors (OSG; ascorbate and dexamethasone); n = 6. Effects of 14 days of MΦ-CM treatment (with OSG), on MSC mRNA levels of (B) Runx2 (C) OSX (D) PTH1R, determined by real-time RT-PCR. Data displayed as mean ± SEM. Statistical significance determined by t-test (B,C,D) or One-Way ANOVA (Tukey's *post hoc* test), (A, *p≤0.05, **p≤0.01, ***p≤0.001 difference from untreated controls or as indicated with capped line; n = 3 or as indicated. Control  = 50% medium conditioned in the absence of cells.

Cell metabolism levels in MSC cultures were raised by the presence of osteogenic medium but were otherwise unaffected by addition of 50% MΦ-CM or OC-CM in cultures (Fig. 3A), suggesting such CM has little or no overall effect on cell growth or activity (Fig. 2D). Nevertheless, after 14 days incubation (in the presence of base osteogenic medium) the presence of either MΦ-CM or OC-CM addition greatly increased the levels of mineralization in the MSC, by five-fold and three-fold respectively (Fig. 3B,C, Fig. S1). In such cultures ALP levels were also strongly elevated (Fig. 4A), consistent with enhanced osteoblastic differentiation. In contrast, conditioned medium from M-CSF-starved macrophages did not induce mineralisation in MSC (data not shown). Surprisingly, we noted that the presence of osteogenic medium (i.e., medium containing ascorbate, dexamethasone and β-glycerophosphate) was not necessary for the pro-osteoblastic actions of MΦ-CM and OC-CM. Omission of osteogenic components from the culture medium resulted in lower levels of MSC ALP levels after 14 days, but ALP levels were still greatly enhanced by the conditioned media (Fig. 4A); indeed the responses to CM were similar to those of cultures in osteogenic medium in terms of fold change relative to baseline. Thus, pro-osteoblastic actions of these conditioned media were not dependent on osteogenic supplements and may arise through a different mechanism. Since MΦ-CM was able to elicit the highest levels of osteoblastic differentiation in MSC we investigated how quickly this occurred compared to conventional 14-day osteogenic treatment. As high ALP levels were induced within 4 days (Fig. S1) this culture period was employed in the ALP analyses below.

To further characterise the effects of MΦ secreted factors on MSC osteoblastic commitment we investigated the expression of Runx2 and osterix (transcription factors critical for osteoblastic commitment and differentiation), as well as the classical PTH receptor (PTH1R) and bone sialoprotein (BSP) which are characteristically expressed by mature osteoblasts. Indeed, MΦ-CM treatment of MSC caused mRNA levels these four factors to rise significantly (Fig. 4B,C,D and Fig. S1C), consistent with enhanced osteoblastic maturation.

### Osteoblastic stimuli produced by CFU-GM-derived macrophages and osteoclasts act in a gp130- and OSM-dependent manner

**Figure 5:**
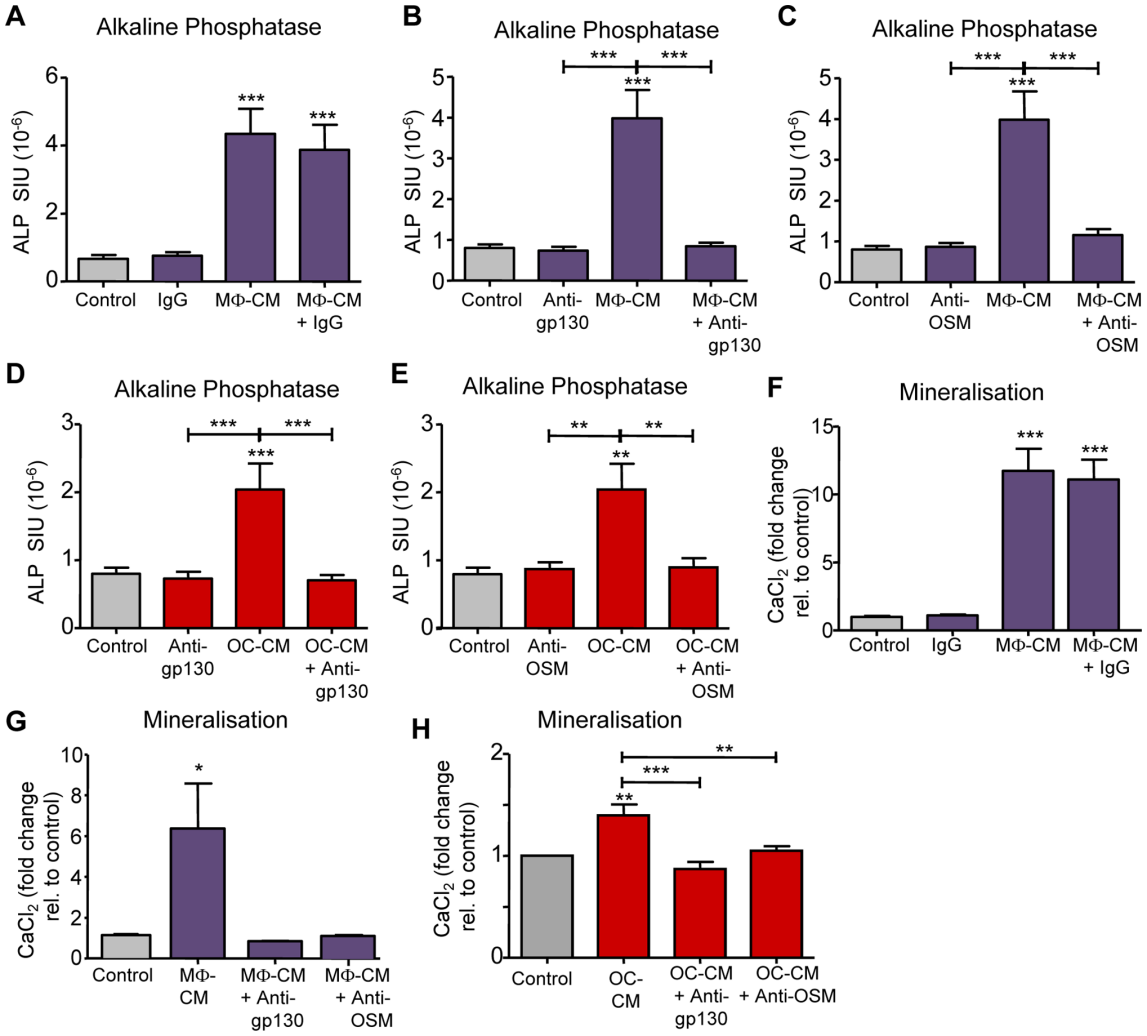
The effects of MΦ-CM and OC-CM on osteoblastic maturation of MSC are gp130 and OSM dependent. MSC were cultured in either 50% MΦ-CM (A-C, F,G) or OC-CM (D, E, H) in osteogenic media in the presence of anti-gp130 (1 µg/mL) or anti-OSM (10 µg/mL) antibodies or IgG (10 µg/ml; control) as indicated. MSC were cultured for 4 days and assessed for ALP activity (A-E), or cultured for 10 days and mineralisation assessed by Alizarin Red binding assay (F-H). Data displayed as mean ± SEM; statistical significance determined by one-way ANOVA (Tukey's *post hoc* test), all data n = 4; *p≤0.05 **p≤0.01, ***p≤0.001 relative to controls (grey columns) or as indicated by capped lines.

We investigated the involvement of gp130-dependent cytokines on the pro-osteoblastic effects of MΦ-CM and OC-CM using anti-gp130 and anti-OSM monoclonal antibodies. Increased ALP levels induced by MΦ-CM treatment of MSC cultures for 4 days was abolished by anti-gp130 antibody (1 µg/mL) but unaffected by control IgG1 (Fig. 5A,B). Anti-OSM (10 µg/mL) also completely abolished the MΦ-CM-elicited rise in ALP (Fig. 5C). Increases in ALP elicited by OC-CM treatment were similarly abolished by anti-gp130 and anti-OSM antibodies (Fig. 5D, E). Consistent with these observations, induction of matrix mineralisation by MΦ-CM and OC-CM treatment of MSC over 14 days was also blocked by anti-gp130 and by anti-OSM antibodies (Fig. 5F, G, H) further confirming that this phenomena are OSM-dependent.

### Production of OSM by CFU-GM-derived macrophages and osteoclasts, and by MSC

**Figure 6:**
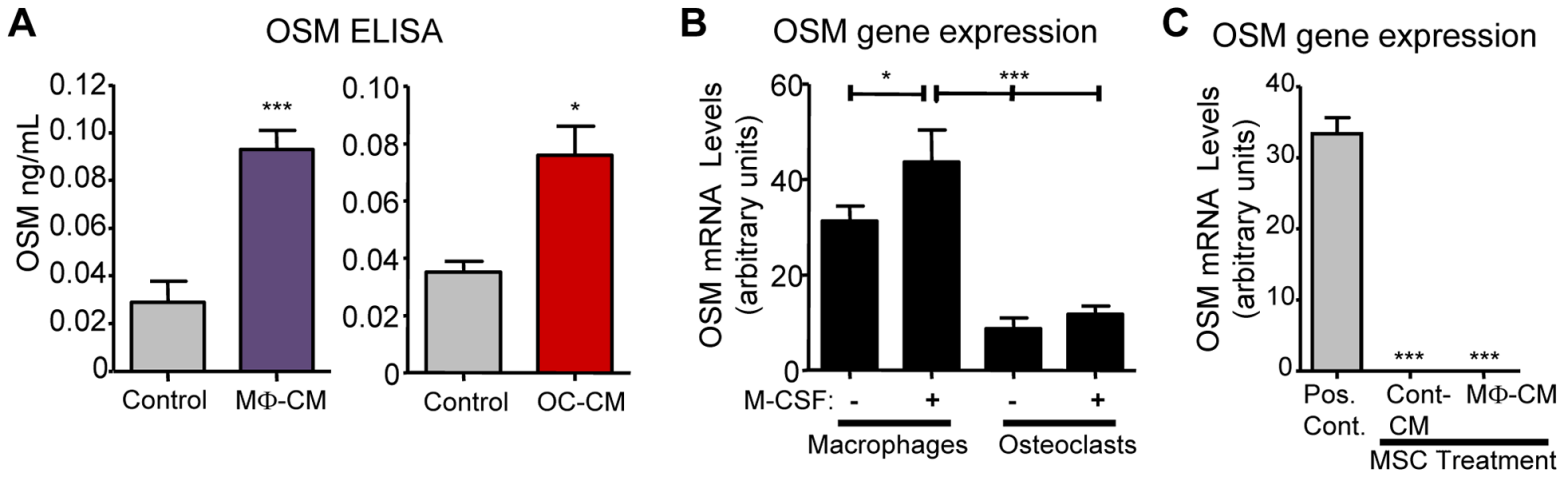
OSM production by macrophages, osteoclasts and MSCs. (A) Levels of OSM protein in MΦ-CM and OC-CM assessed by ELISA. (B) Relative OSM gene expression in macrophage and OC cultures assessed by RT-PCR. (C) Lack of OSM mRNA detection in CM-treated (4 days) MSC cultures; ‘Pos. Cont’ shows macrophage mRNA data as a method positive control. Data displayed as mean ± SEM and statistical significance relative to controls (or as indicated by capped bars) determined by one-way ANOVA (Tukey's *post hoc* test) or Student's t-test, n = 4, *p≤0.05, ***p≤0.001 or as indicated by capped lines.

Having shown that the effects of MΦ-CM and OC-CM were OSM dependent, we determined by ELISA methods that OSM was indeed detectable in MΦ-CM and in OC-CM (Fig. 6A); RT-PCR analysis also confirmed that our macrophage and osteoclast populations expressed OSM mRNA (Fig. 6B) although the presence of M-CSF itself did not have a large effect on OSM mRNA levels.

In our differentiating MSC cultures, the added conditioned medium is not the only possible source of OSM, since the MSC themselves may produce it, indeed, MΦ-CM may contain factors that induce OSM production in MSC cultures. However, by RT-PCR methods OSM mRNA was undetectable in the MSC and did not become evident with MΦ-CM treatment (Fig. 6C). Further to this, medium taken from MSC cultures after 4 days of incubation contained very low (indeed, barely detectable) levels of OSM protein as did medium from MSC exposed to MΦ-CM for this period (Fig. S2A). These observations suggest autocrine actions of MSC-derived OSM are unlikely to contribute to osteoblastic differentiation responses in MΦ-CM-stimulated MSC cultures.

### The effects of recombinant OSM on MSC cultures, and OSM interactions with osteogenic factors BMP-2 and Wnt3A

**Figure 7:**
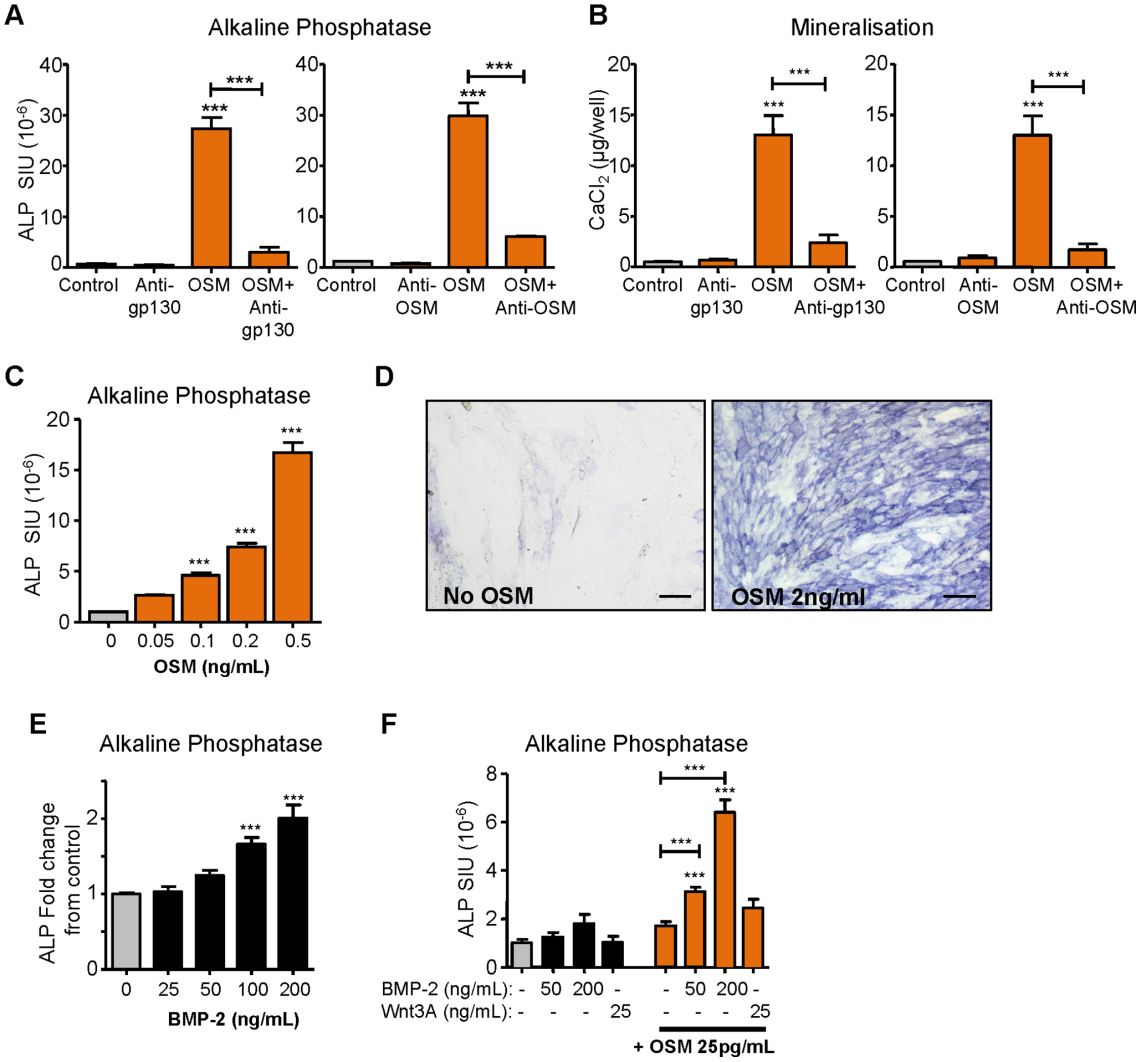
The effects of OSM on osteoblastic maturation of MSC. To confirm OSM actions, MSC were cultured in osteogenic media with recombinant OSM (10 ng/mL) for 4 days and assessed for (A) ALP activity, or for 10 days and assessed for (B) mineralisation, by Alizarin Red binding assays; blocking of recombinant OSM actions by antibodies to gp130 and OSM was confirmed; n = 4. (C) OSM dose response of MSC ALP levels (at day 4) and (E) photomicrographs ALP histochemical stain in control and OSM treated MSC, day 4; bars  = 100 µm. (E) BMP-2 dose response of MSC ALP levels, day 4. (F) Synergistic actions of OSM with BMP-2 but not Wnt3A co-treatment on MSC ALP levels at 4 days of incubation (with osteogenic factors). Data displayed as mean ± SEM and statistical significance relative to controls (grey columns), or as indicated by capped bars, determined by one-way ANOVA (Tukey's *post hoc* test), n = 3 unless noted, *p≤0.05 **p≤0.01, ***p≤0.001.

Since OSM activity is essential for the osteoblastic actions of MΦ-CM and OC-CM on our human adipose tissue MSC, we confirmed that, as previously described, [32] 10 ng/mL recombinant human OSM strongly induces ALP and mineralisation in MSC cultures (Fig. 7A,B). However, our ELISA data in Fig. 6 indicated that the OSM levels in MΦ-CM and OC-CM were very low, around 0.1 ng/ml. We thus tested the effects of recombinant OSM levels in this range on MSC ALP levels. We found that 0.1 ng/ml recombinant OSM was indeed sufficiently potent to greatly increase these ALP levels, with an almost 5-fold increase (observed over 4 days) and higher ALP levels with greater OSM concentrations (Fig. 7C,D); matrix mineralisation was also significantly increased by 0.2 ng/ml OSM (Fig. S2B). Since OSM is likely to be found in microenvironments where other osteogenic factors are present we examined the interaction of low concentrations of OSM with BMP-2 and Wnt3A on MSC cultures; note that we did not find significant BMP or Wnt activity in MΦ-CM itself using luciferase reporter assays (Fig. S2C,D). BMP-2 100 ng/ml significantly induced our adipose tissue MSC ALP levels (4 day cultures; Fig. 7E), though much less potently than OSM; Wnt3A treatment had no detectable effects (Fig. S2E). Co-treatment with 50 ng/ml BMP-2 and 0.025 ng/ml OSM (both concentrations without significant effects on MSC) caused a sizable increase in ALP levels, suggesting synergistic interactions between these two factors (Fig. 7F). The effects of 2 ng/ml OSM also showed significant enhancement by 50 ng/ml and 100 ng/ml BMP-2 (Fig. S2F). Recombinant Wnt3A did not detectably influence OSM actions on MSC (Fig. 7F).

### The effects of activation on CFU-GM-derived macrophage pro-osteoblastic stimulation of MSC

**Figure 8:**
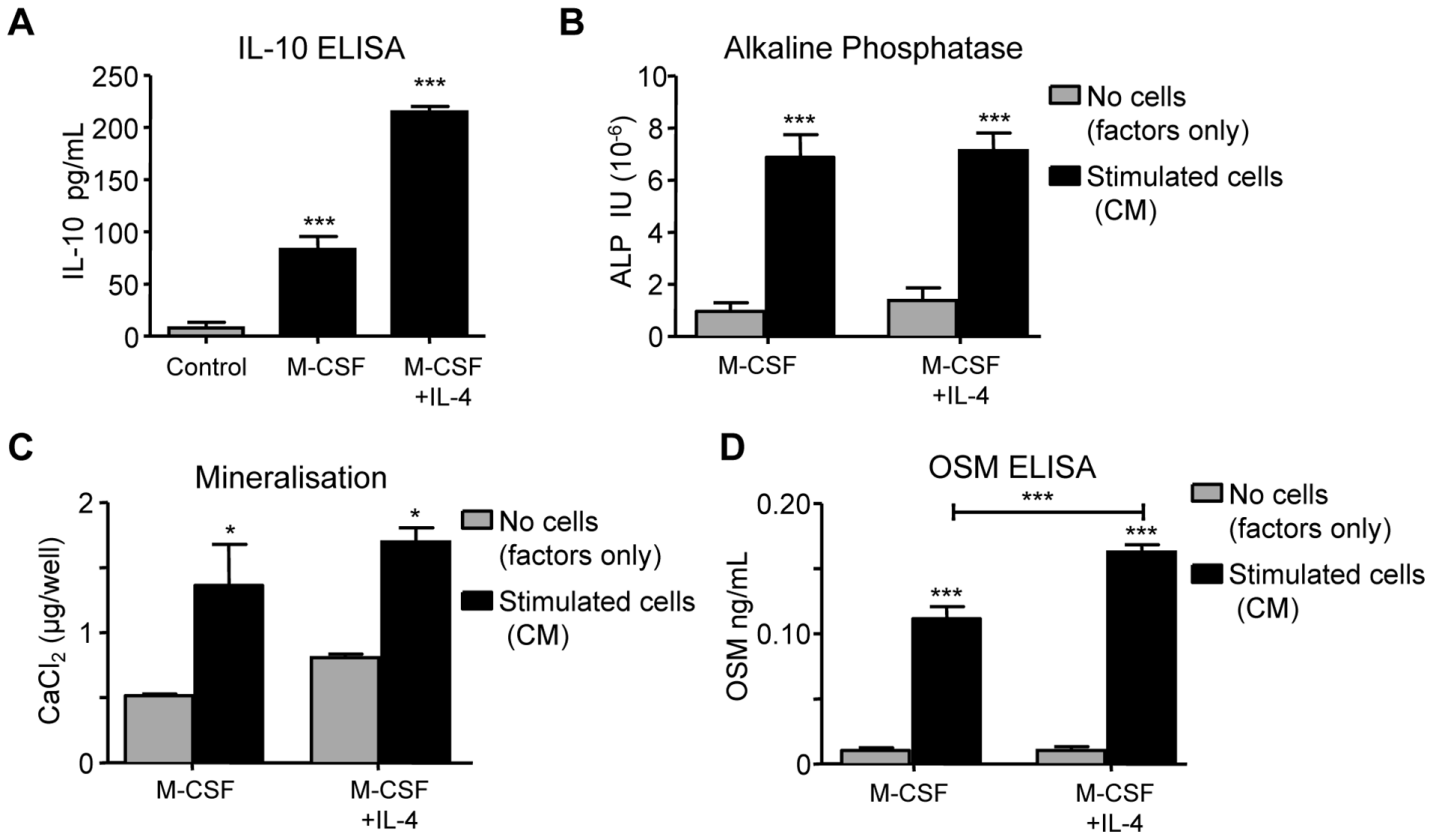
The influence of IL-4 on CFU-GM-derived macrophage stimulation of MSC osteoblastic differentiation. CM was generated from macrophages cultured in M-CSF (25 ng/mL) or M-CSF plus IL-4 (100 ng/mL) for 14 days, and medium conditioned in these cells for a further 3 days. (A) IL-10 levels in CM determined by ELISA; ‘Control’  =  medium (with M-CSF) conditioned without cells. (B) MSC were exposed to CM for 4 days and assessed for ALP activity or (C) exposed to CM for 14 days and mineralisation assessed by staining by Alizarin Red binding assays. (D) Levels of OSM in CM assessed by ELISA. Data displayed as mean ± SEM; statistical significance determined by one-way ANOVA (Tukey's *post hoc* test), n = 4, **p≤0.01, ***p≤0.001 compared to controls (grey columns) or as indicated (capped line).

To investigate the influence of macrophage activation we first investigated the effect of alternative activation of macrophages using IL-4 (10 ng/mL) treatment applied for 3 days. IL-4 treatment of the macrophages for 3 days did not elicit TNF or IL-1β levels but, consistent with alternative activation, significantly increased levels of human IL-10 produced (Fig. 8A). IL-4 treatment of macrophages did not significantly affect the ability of CM generated from these cells to enhance MSC ALP expression and matrix mineralization (Fig. 8B,C). OSM levels in CM generated from IL-4-treated cells were slightly greater than positive controls (Fig. 8D), although we could not detect any difference in OSM mRNA expression between M-CSF- and M-CSF+IL-4-treated macrophages (data not shown).

**Figure 9:**
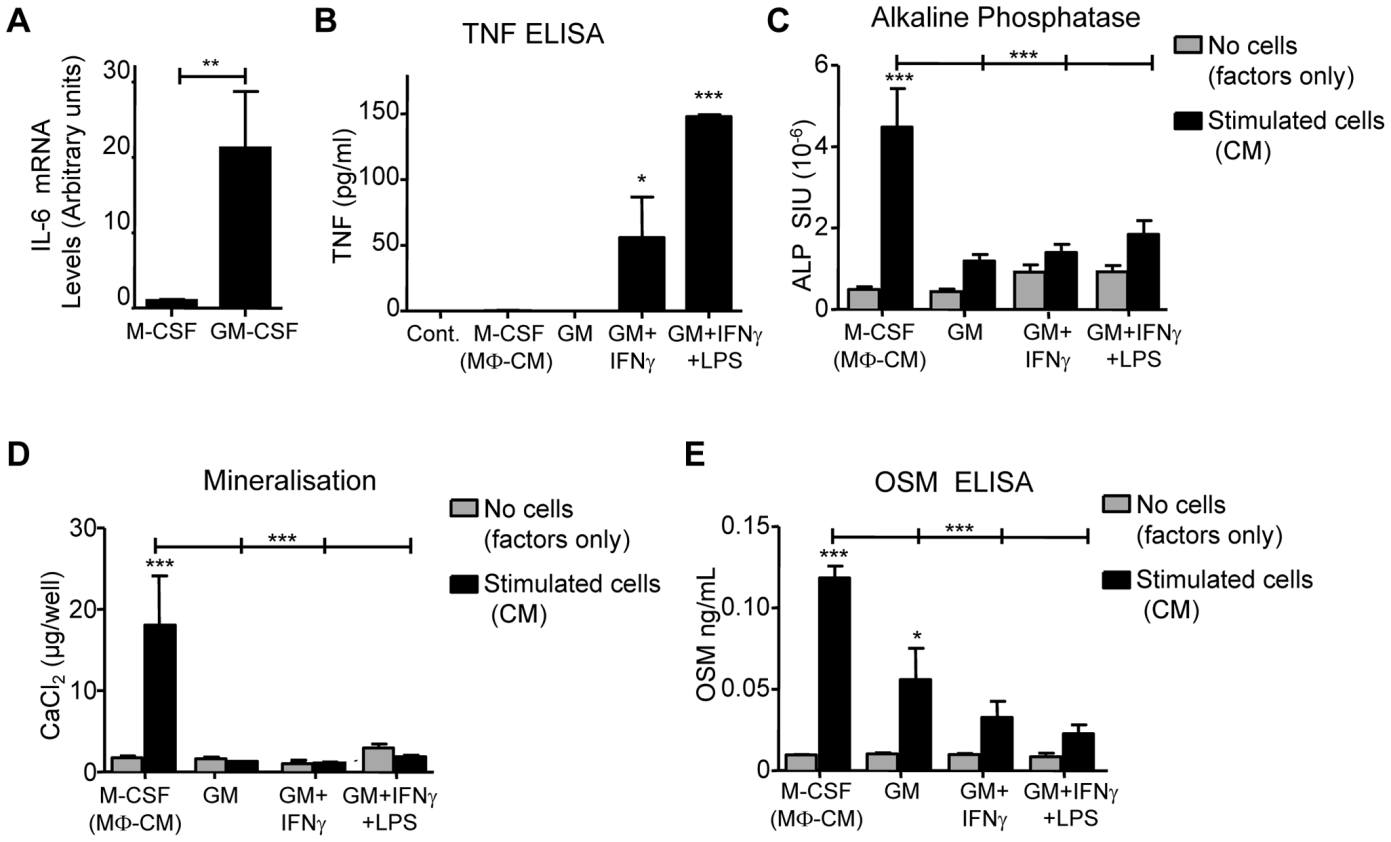
The effects of classical activation on the ability of macrophages to stimulate osteoblastic commitment of MSC. CFU-GM populations were cultured in GM-CSF (10 ng/mL) for 14 days, then stimulated with the following: GM-CSF (‘GM’) alone; GM-CSF plus IFNγ (“GM+IFNγ”); or GM-CSF plus IFNγ followed by 3 days in GM-CSF plus LPS (“GM+IFNγ+LPS”). (A) Higher IL-6 and TNF mRNA expression in macrophages generated in GM-CSF than M-CSF (determined by RT-PCR). (B) TNF protein was determined (by ELISA) in conditioned medium from M-CSF, GM, GM+IFNγ and GM+IFNγ+LPS treated macrophages. ‘Cont.’  =  medium (containing GM-CSF) conditioned without cells. (C) MSC were exposed to CM from the cells indicated and MSC cultures assessed for ALP activity after 4 days and (D) mineralisation (Alizarin Red binding assay) at 14 days. (E) Levels of OSM in CM assessed by ELISA. Data displayed as mean ±SEM; statistical significance determined by one-way ANOVA (Tukey's *post hoc* test); A,B n = 3, C-E n = 4. *p≤0.05, **p≤0.01, ***p≤0.001 compared to their controls (in adjoining grey columns) or as indicated by capped lines.

To investigate the effects of classical activation, CFU-GM-derived cells were expanded with GM-CSF 10 ng/mL (rather than M-CSF) for 14 days (GM-MΦ); some GM-CSF expanded cells were also further activated by treatment with IFNγ (1 ng/mL) or a combination of IFNγ plus 100 ng/mL LPS (Fig. 9). Both GM-CSF and M-CSF expansion produced cell cultures of similar cell density after 14 days. GM-CSF-expanded cells (GM-MΦ) expressed higher levels of IL-6 mRNA than M-CSF expanded cells (Fig. 9A). IL-1β, IL-10 or TNF were not detectable in conditioned medium of these cells but activation by IFNγ or IFNγ plus LPS significantly induced TNF production (Fig. 9B). These IFNγ plus LPS-activated cell populations analysed by FACS had far fewer CD16^+^ cells (1.01±0.99%) than M-CSF-expanded macrophage populations (Table 1); the IFNγ plus LPS-activated cell contained no CD14^+^/CD16^+^ and very few CD14^+^/CD206^+^ cells (2.0±0.90%), while CD14^+^/CD86^+^ were numerous (26.29±0.89% of cells).

The CM from these activated macrophage-containing populations (GM-MΦ, GM+ IFNγ-MΦ and GM+ IFNγ+LPS-MΦ) all failed to increase MSC ALP and mineralisation levels in MSC relative to negative controls (Fig. 9C,D). OSM levels in medium conditioned by these activated macrophage populations were also lower than medium conditioned by M-CSF-treated macrophages (MΦ-CM; Fig. 9E).

## Discussion

One of the most significant puzzles in bone biology is how osteoblast formation and activity is controlled in normal healthy bone and in disease states. The need for improved anabolic therapies for bone makes identifying the mechanisms that underlie osteoblast recruitment an important goal. Currently the main bone anabolic therapy that is employed clinically is injected PTH. This treatment stimulates bone formation through several mechanisms, including enhancement of osteoblast survival and reduction of sclerostin production by osteocytes [12]. However, PTH anabolic action can be blunted by anti-resorptive drugs that reduce osteoclast number [12], [33]. This, and the coupling of resorption and formation in bone remodelling suggest that osteoclasts stimulate osteoblast action, although how this may occur is not understood. We have presented here evidence suggesting that human osteoclastic cells stimulate osteoblastic differentiation of MSC. However, this capacity was shared by macrophage populations from which our osteoclasts were derived, showing that it is not osteoclast specific. Nevertheless, our work (and that of Guihard et al [22]) suggest that not all types of macrophages have this action and those with a close ontogenic relationship with osteoclasts may be particularly pro-osteoblastic. We found that this activity was not due to production of bone morphogenetic protein (BMP) or Wnt activity (two well characterised factors that drive osteoblast differentiation), but depended entirely on macrophage or osteoclast production of OSM, an IL-6 cytokine family member.

OSM is an anabolic factor for bone *in vivo* that, in addition to its effects on MSC [32], suppresses osteocytes production of sclerostin, [34], a Wnt-inhibiting factor that is under active study as a therapeutic target. We previously found that OSM has strong bone anabolic effects in mice *in vivo*, but OSM can be produced by a number of local cell types, including mature osteoblasts themselves [34], [35] and it is unclear to what degree OSM directly causes murine MSC maturation. While an OSM-dependent action of osteoclasts and macrophages may be significant for MSC recruitment it is unlikely to explain how bone formation is stimulated by osteoclast-initiated remodelling unless other factors either direct MSC to the site of osteoclast activity or provide an amplifying co-stimulus for OSM action. In this regard it is also notable that mice deficient in the OSM receptor do not have disordered bone formation [34]; OSM null mice have been also been studied, particularly for haematopoiesis defects [36] but no gross bone abnormality reported. We previously found that mice lacking gp130-SHP2/ERK signalling (but not gp130-STAT3 signals) do have reduced bone mass but not disordered remodelling [37]. Clearly, OSM cannot therefore be essential for normal murine bone remodelling, although its actions are likely to overlap with other related cytokines [38] and interact with other osteogenic factors, as we found with BMP-2. These considerations, and the fact that the degree of MSC recruitment in normal bone modelling and remodelling is unclear, currently makes it difficult to assess the importance of OSM in bone metabolism.

Our data is clearly consistent with an influence of macrophages on MSC, but the nature of macrophage influence on bone formation remains controversial. Striking observations were made by Alexander *et al.* [16] in a fracture model where they noted that depletion of c-fms^+^ cells (principally macrophages, but also osteoclasts) greatly reduced osteoblast numbers and bone formation. Due to the location of resident macrophages near the bone surface and the effects of macrophage depletion they proposed a central role for macrophages in enhancing or maintaining bone formation, involving osteal macrophages in close contact with osteoblasts [14]. Given the many varied functions of macrophages and the abundance of macrophages in bone and bone marrow, this is certainly plausible. It also suggests that factors that stimulate or recruit macrophages could indirectly influence bone formation, and a role here for OSM is possible. Furthermore, we noted that not only was OSM extremely potent in its effects on MSC but it acted synergistically with BMP-2. The possibility that OSM cooperates with this and other factors to increase bone formation, in addition to the ability of OSM to suppress sclerostin and induce production of osteoblastic factors like IL-33 [39], suggest that further scrutiny of macrophage (and OSM) actions in bone are warranted.

The work of Guihard *et al.* [22] and more recently Nicolaidou *et al.* [23] identified that MSC (derived from human bone marrow stroma) also undergo enhanced osteoblastic differentiation in response to mature macrophages derived from human CD14^+^ circulating monocytes; this occurred in a manner at least partly dependent on OSM. The conclusions of these studies otherwise differ markedly in many respects, and differ in some respects to our study. Guihard *et al.* [22] found that conditioned medium from GM-CSF- and IFNγ-stimulated monocyte-derived CD14^+^ cells (classically activated macrophages) drive osteoblastic differentiation of MSC, and that this is enhanced by LPS treatment, while conditioned medium from CD14^+^ monocytes treated with alternative activators IL-4 or IL-10 did not. In contrast, Nicolaidou *et al*. [23] found that medium conditioned by human monocytes did not drive MSC osteoblastic differentiation at all unless co-cultured with the MSC; consistent with this, conditioned medium from monocyte/MSC co-cultures stimulated osteoblast maturation in other MSC cultures. In our work we employed proliferating macrophages derived from progenitors that resemble immature bone marrow macrophages rather more than adult monocytes; our M-CSF-treated cord blood-derived macrophages expressed CD14 but also CD16, suggesting a ‘non classical’ monocyte phenotype [30]. Classical activation of these cells (which reduced CD16 expression) resulted in lower OSM levels, also indicating that these macrophages differ markedly from adult monocytes employed by Guihard *et al.* We found that cord blood-derived macrophages directly co-cultured with MSC induced only a weak action on ALP expression compared to MΦ-CM (data not shown). We are uncertain why this is the case, but it may be due to technical aspects that require further study, as these immature macrophages did not appear to thrive in such co-cultures. Our studies also indicated that macrophages treated with either M-CSF or M-CSF plus IL-4 produced strong (OSM-dependent) osteoblastic stimuli. This indicates that alternative macrophage activation maintained their pro-osteoblastic activity, again unlike the CD14^+^ adult monocyte derived populations studied by Guihard *et al.* [22]. In general, these data collectively suggest there may be significant differences between our CFU-GM-derived macrophages and monocyte-derived macrophages. It seems reasonable to assume that a pro-osteoblastic or anti-osteoblastic outcome of a given stimulus could depend greatly on features of the macrophage populations. This suggests that determining the profile of macrophages present in bone and bone lesions (which would presumably include both monoctyes and CFU-GM) is critical for understanding the outcome of a particular stimulus. A detailed comparison of our experimental system with that used by Guihard *et al*. [22] and Nicolaidou *et al* [23] may also clarify how macrophages affect bone formation. It is notable that classically activated macrophages *in vivo* are associated with chronic inflammation and low bone formation, and since inflammation commonly drives osteolysis this can make inflammatory lesions very destructive to bone. However, inflammation can have a pro-osteoblastic outcome, as commonly observed in ankylosing spondylitis. The reasons for this are unclear but properties of the recruited macrophages in these lesions could play an influential role. Such contrasting effects on local bone metabolism can also be seen in cancer invasion – for example, although breast cancers are typically osteolytic, osteoblastic lesions also occur.

In summary, we have found that macrophage- and osteoclast-derived OSM stimulates MSC differentiation to osteoblasts. This stimulation occurs more rapidly (within 4 days of incubation), than widely used ascorbate/dexamethasone treatment, making this a very useful experimental system. This OSM-mediated interaction, consistent with the observations of Song *et al*. [32], may play a significant role in stimulating and maintaining osteoblastic activity in bone, at least where this dependent on MSC differentiation. We found that immature M-CSF-dependent macrophages (which are abundant in bone marrow), including those exposed to IL-4 to induce alternative activation, are a good source of this pro-osteoblastic activity. However this activity is significantly reduced or abolished by GM-CSF exposure or classical activation, although whether this is solely due to their reduced OSM production or whether they produce anti-osteoblastic factors is unclear. This work confirms that macrophages and osteoclasts can have a major effect on osteoblastic cell recruitment from MSC, although the specific phenotype of the macrophages present is clearly important.

## Supporting Information

Figure S1 **Time course of MΦ-CM effects on MSC maturation.** MSC were cultured in medium containing osteogenic factors (ascorbate, dexamethasone and β–glycerophosphate) for 4, 7 and 14 days, with or without addition of 50% MΦ-CM as indicated, then assessed for (A) ALP activity and (B) mineralisation. Data displayed as mean ± SEM; statistical significance determined by one-way ANOVA (Tukey's *post hoc* test), n = 4, **p≤0.01 and ***p≤0.001 compared to respective control cultures. (C) BSP and GAPDH mRNA levels were examined (by semi-quantitative RT-PCR) in MSC cultured for 14 days with osteogenic factors alone (Control) or with addition of 50% MΦ-CM; representative of 3 independent cultures.
(PDF)

Figure S2 **The lack of production of OSM by MSC, BMP and Wnt activity in MΦ-CM, and the influence of BMP-2, Wnt3A and OSM on MSC maturation.** (A) MSC were cultured in 50% MΦ-CM or control medium (‘Cont-Med’) for 4 days. OSM levels in the resulting MSC-exposed culture medium were assessed by ELISA but showed only very low levels, much lower than MΦ-CM (‘Pos. Cont.’) alone. (B) Detailed dose response of MSC matrix mineralisation (at day 7) to OSM treatment. To detect BMP and Wnt protein activity in MΦ-CM (50%), luciferase reporter-based assays were employed, using BMP-RE and TOPFlash reporters respectively. (C) UMR106.01 osteoblastic cells transiently co-transfected with BMP-RE luciferase and Renilla reporter constructs, 24h incubation; ‘Cont.’  =  control medium conditioned without cells, BMP-2  = 100 ng/mL. (D) UMR106.01 cells were used as in B, but with TOPflash luciferase constructs and Renilla reporter construct; Wnt3A  = 100 ng/mL. (E) Lack of effects on ALP responses of Wnt3A (100 ng/mL) after 4 days of incubation. (F) Co-operative actions of 2 ng/ml OSM with BMP-2 (but not Wnt3A) co-treatment on MSC ALP levels at 4 days of incubation with osteogenic factors; n = 3. Data displayed as mean ± SEM; statistical significance determined by one-way ANOVA (Tukey's *post hoc* test), all n = 3. *p≤0.05, **p≤0.01 and ***p≤0.001 compared to control cultures (grey columns).
(PDF)
