# Supplementary figures and images for: Cord Blood-Derived Macrophage-Lineage Cells Rapidly Stimulate Osteoblastic Maturation in Mesenchymal Stem Cells in a Glycoprotein-130 Dependent Manner
Source: PLoS One. 2013 Sep 12;8(9):e73266. doi: 10.1371/journal.pone.0073266 (PMC3772005; doi:10.1371/journal.pone.0073266)

Supporting Figure S1

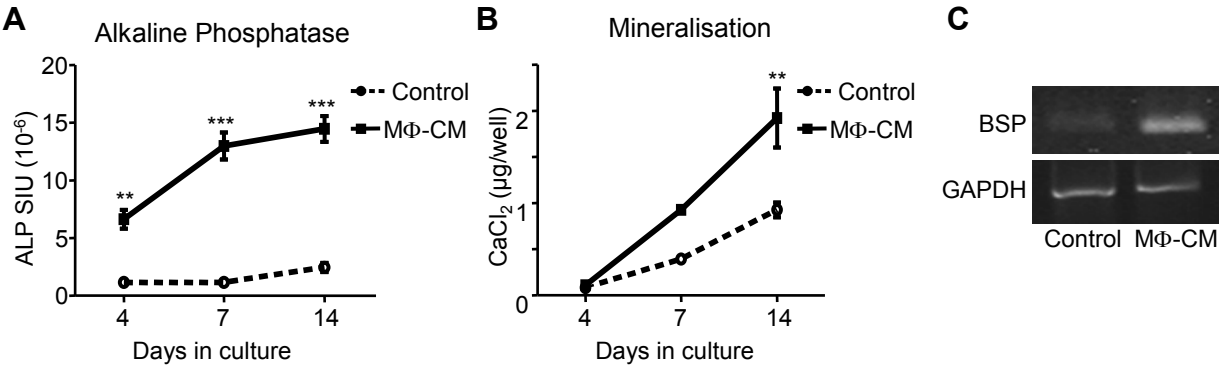

Supplement: Figure S1 — Time course of MΦ-CM effects on MSC maturation. MSC were cultured in medium containing osteogenic factors (ascorbate, dexamethasone and β–glycerophosphate) for 4, 7 and 14 days, with or without addition of 50% MΦ-CM as indicated, then assessed for (A) ALP activity and (B) mineralisation. Data displayed as mean ± SEM; statistical significance determined by one-way ANOVA (Tukey's post hoc test), n = 4, **p≤0.01 and ***p≤0.001 compared to respective control cultures. (C) BSP and GAPDH mRNA levels were examined (by semi-quantitative RT-PCR) in MSC cultured for 14 days with osteogenic factors alone (Control) or with addition of 50% MΦ-CM; representative of 3 independent cultures. (PDF) [file pone.0073266.s001.pdf]

Supporting Figure S2

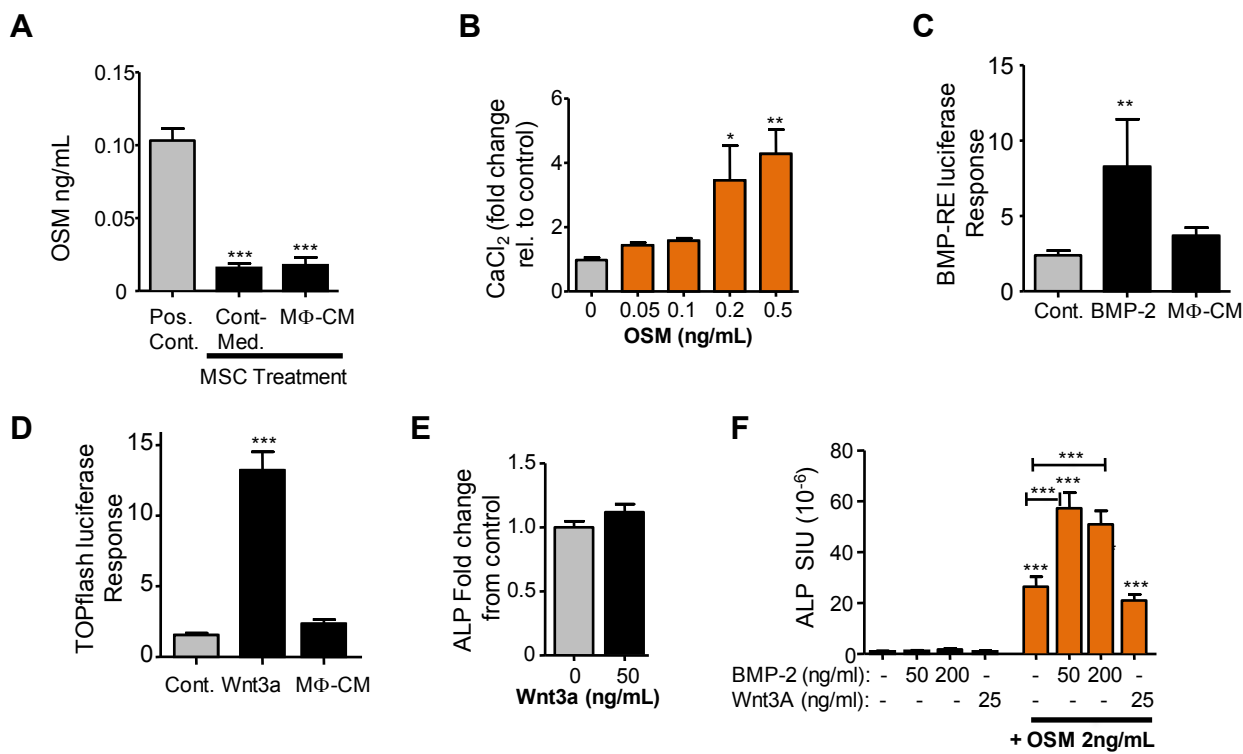

Supplement: Figure S2 — The lack of production of OSM by MSC, BMP and Wnt activity in MΦ-CM, and the influence of BMP-2, Wnt3A and OSM on MSC maturation. (A) MSC were cultured in 50% MΦ-CM or control medium (‘Cont-Med’) for 4 days. OSM levels in the resulting MSC-exposed culture medium were assessed by ELISA but showed only very low levels, much lower than MΦ-CM (‘Pos. Cont.’) alone. (B) Detailed dose response of MSC matrix mineralisation (at day 7) to OSM treatment. To detect BMP and Wnt protein activity in MΦ-CM (50%), luciferase reporter-based assays were employed, using BMP-RE and TOPFlash reporters respectively. (C) UMR106.01 osteoblastic cells transiently co-transfected with BMP-RE luciferase and Renilla reporter constructs, 24h incubation; ‘Cont.’ = control medium conditioned without cells, BMP-2 = 100 ng/mL. (D) UMR106.01 cells were used as in B, but with TOPflash luciferase constructs and Renilla reporter construct; Wnt3A = 100 ng/mL. (E) Lack of effects on ALP responses of Wnt3A (100 ng/mL) after 4 days of incubation. (F) Co-operative actions of 2 ng/ml OSM with BMP-2 (but not Wnt3A) co-treatment on MSC ALP levels at 4 days of incubation with osteogenic factors; n = 3. Data displayed as mean ± SEM; statistical significance determined by one-way ANOVA (Tukey's post hoc test), all n = 3. *p≤0.05, **p≤0.01 and ***p≤0.001 compared to control cultures (grey columns). (PDF) [file pone.0073266.s002.pdf]
